# The effect of transdermal scopolamine for the prevention of postoperative nausea and vomiting

**DOI:** 10.3389/fphar.2014.00055

**Published:** 2014-04-09

**Authors:** María A. Antor, Alberto A. Uribe, Natali Erminy-Falcon, Joseph G. Werner, Keith A. Candiotti, Joseph V. Pergolizzi, Sergio D. Bergese

**Affiliations:** ^1^Department of Anesthesiology, Perioperative Medicine and Pain Management, Jackson Memorial HospitalMiami, FL, USA; ^2^Department of Anesthesiology, The Ohio State University Werner Medical CenterColumbus, OH, USA; ^3^Department of Internal Medicine, Riverside Methodist HospitalColumbus, OH, USA; ^4^Department of Medicine, The Johns Hopkins University School of MedicineBaltimore, MD, USA; ^5^Department of Neurological Surgery, The Ohio State University Medical CenterColumbus, OH, USA

**Keywords:** nausea, vomiting, antiemetic, postoperative, prophylaxis, transdermal scopolamine, pharmacokinetics, pharmacodynamics

## Abstract

Postoperative nausea and vomiting (PONV) is one of the most common and undesirable complaints recorded in as many as 70–80% of high-risk surgical patients. The current prophylactic therapy recommendations for PONV management stated in the Society of Ambulatory Anesthesia (SAMBA) guidelines should start with monotherapy and patients at moderate to high risk, a combination of antiemetic medication should be considered. Consequently, if rescue medication is required, the antiemetic drug chosen should be from a different therapeutic class and administration mode than the drug used for prophylaxis. The guidelines restrict the use of dexamethasone, transdermal scopolamine, aprepitant, and palonosetron as rescue medication 6 h after surgery. In an effort to find a safer and reliable therapy for PONV, new drugs with antiemetic properties and minimal side effects are needed, and scopolamine may be considered an effective alternative. Scopolamine is a belladonna alkaloid, α-(hydroxymethyl) benzene acetic acid 9-methyl-3-oxa-9-azatricyclo non-7-yl ester, acting as a non-selective muscarinic antagonist and producing both peripheral antimuscarinic and central sedative, antiemetic, and amnestic effects. The empirical formula is C_17_H_21_NO_4_ and its structural formula is a tertiary amine L-(2)-scopolamine (tropic acid ester with scopine; *MW* = 303.4). Scopolamine became the first drug commercially available as a transdermal therapeutic system used for extended continuous drug delivery during 72 h. Clinical trials with transdermal scopolamine have consistently demonstrated its safety and efficacy in PONV. Thus, scopolamine is a promising candidate for the management of PONV in adults as a first line monotherapy or in combination with other drugs. In addition, transdermal scopolamine might be helpful in preventing postoperative discharge nausea and vomiting owing to its long-lasting clinical effects.

## INTRODUCTION

Postoperative nausea and vomiting (PONV) are among the most common complaints from patients and clinicians (Jones et al., 2006; Sood et al., 2007). Prevention and treatment of PONV is a key patient care component capable of alleviating patient discomfort, distress, and dissatisfaction during the postoperative period. PONV *per se* is a significant complication that may lead to other postoperative adverse events including aspiration, pneumonitis, dehydration, wound dehiscence, acid-base disorders, and electrolyte imbalance, hematoma formation, esophageal rupture, increases in intraocular and/or intracranial pressures, and acute blood pressure elevations (Fabling et al., 2000; Lipp and Kaliappan, 2007; Feng et al., 2009). According to Society of Ambulatory Anesthesia (SAMBA) Consensus Guidelines for the Management of PONV, the incidence of PONV is between 20–30% among all patients undergoing surgery. With no prophylactic therapy, PONV occurs in as many as 70–80% of high-risk patients undergoing surgery (Gan et al., 2014). Three categories of baseline risk factors: patient-specific, anesthetic, and surgical, are independent predictors of PONV, and may be helpful in selecting the right candidates for prophylaxis. The most prevalent patient and anesthesia related risk factors for PONV are female gender, non-smoking status, postoperative opioid consumption, and a history of PONV/postoperative discharge nausea and vomiting (PDNV) or motion sickness (Apfel et al., 1999). Each factor has a punctuation value of 1, which, when added, will equal 0–4. When 0, 1, 2, 3, or 4 of the above mentioned independent predictors are present; the corresponding risk for PONV is approximately 10, 20, 40, 60, or 80%, respectively (Gan and Habib, 2003; Gan et al., 2014). Presence of a single risk factor corresponds to a 20% risk of PONV, while combination of all the risk factors will be related to an 80% chance of PONV (Apfel et al., 1999). The SAMBA Consensus Guidelines for the Management of PONV include assessing the patient’s risk, reducing the baseline risk factors, and providing prophylactic treatment. The current (Gan et al., 2014) prophylactic therapy recommendations for PONV management stated in the SAMBA guidelines should start with monotherapy and patients at moderate to high risk, a combination of antiemetic medication should be considered. Consequently, if rescue medication is required, the antiemetic drug chosen should be from a different therapeutic class and administration mode than the drug used for prophylaxis. Considering the increasing rate of outpatient surgeries, long-lasting prophylactic antiemetic therapeutic efforts may be beneficial for postoperative patient care and improve the outcomes. Scopolamine is a long-acting prophylactic antiemetic (72 h) approved in 1979 by the Food and Drug Administration (FDA; Apfel et al., 2010) as a method of preventing motion sickness and, since 2001 – for prevention of PONV. In outpatient surgical patients, PDNV usually remains unrecorded because of early discharge, despite the fact that the patients report this complication as the most undesirable postoperative event. Thus, taking into account the beneficial effects of scopolamine, its favorable pharmacokinetic and pharmacodynamic profile, and importance of PONV prophylaxis, transdermal delivery system (TDS) may become a strong candidate as a first-line prophylactic medication in perioperative care (Pergolizzi et al., 2012).

## OVERVIEW OF SCOPOLAMINE

Scopolamine is a non-polar, belladonna alkaloid, α-(hydroxymethyl) benzeneacetic acid 9-methyl-3-oxa-9-azatricyclo non-7-yl ester. The empirical formula is C_17_H_21_NO_4_ and its structural formula is a tertiary amine L-(2)-scopolamine. It is a viscous liquid that has a molecular weight of 303.35 and a pKa of 7.55–7.81. Scopolamine is a high-affinity selective competitive antagonist of G protein-coupled muscarinic receptor for acetylcholine with both, peripheral and central antimuscarinic effects, including sedative, antiemetic, and amnesic action (Pergolizzi et al., 2011). It acts on the central nervous system (CNS) by blocking cholinergic transmission from vestibular nuclei to higher CNS centers and from the reticular formation to the vomiting center (Renner et al., 2005; Package Insert, 2006). Scopolamine is available as an oral tablet, injectable solution, and TDS. However, the oral and/or parenteral routes of administration are rarely used due to pronounced dose-dependent side effects (excessive sedation, agitation, hallucinations, vertigo, dry mouth, and drowsiness) and a short plasma half-life.

## TRANSDERMAL SCOPOLAMINE: PHARMACOKINETICS AND PHARMACODYNAMIC PROPERTIES

Transdermal delivery system (TDS) delivery system (patch) functions as a long-acting prophylactic antiemetic (72 h). It is a 0.2 mm thick and 2.5 cm^2^ film, with four layers (Pergolizzi et al., 2012). The first layer of the patch is a tancolored aluminized polyester film. The second layer is a drug reservoir mixture of scopolamine, light mineral oil, and polyisobutylene. The third layer represents a dose delivery rate controlling microporous polypropylene membrane, and the fourth layer is an adhesive surface formulation of mineral oil, polyisobutylene, and priming dose of scopolamine applied directly to the skin (Nachum et al., 2006; Package Insert, 2006). Only the active drug scopolamine is being released from the delivery system during the TDS application. This patch is designed for continuous release of scopolamine following application to the skin, with the highest permeation rate in the postauricular (mastoid) area, and the lowest permeation in the thigh, higher in the forearm, and still higher in the stomach, chest and back (Pergolizzi et al., 2011).

The TDS contains 1.5 mg of the drug in a reservoir designed to provide a continuous slow release of scopolamine through intact skin during the first 72 h of patch application (Pergolizzi et al., 2011). The priming dose of scopolamine (140 μg), when applied to the post-auricular area, increases the plasma detectable levels are reached within 4 h with a peak level at 24 h. The rate of scopolamine release after the patch application equals 0.5 mg/day of scopolamine over a 3 days period. The average plasma concentration produced is 87 pg/mL for free scopolamine and 354 pg/mL for total scopolamine (free fraction and conjugated drug; Package Insert, 2006). After removal of the used system, plasma scopolamine levels decrease gradually with an elimination half-life of 9.5 h (Pergolizzi et al., 2011). Upon absorption, scopolamine is mostly bound to plasma proteins and undergoes an almost complete hepatic elimination via hepatic conjugation with subsequent urinary excretion of hydrophilic metabolites. Less than 5% of scopolamine is excreted unchanged (Renner et al., 2005; Nachum et al., 2006). Like atropine, scopolamine acts as a non-specific competitive antagonist of acetylcholine at muscarinic receptors. Due to receptor sensitivity, the inhibition of salivation (M3 receptors) can be reached at lower doses, whereas much higher doses are needed to induce cardiac effects (M2 receptors). As a result, the measured plasma scopolamine concentration does not necessarily correlate with the extent of pharmacodynamic effects of the drug. Scopolamine pharmacodynamics only quantitatively differs from that of atropine. Whereas atropine has almost no detectable effects on CNS in clinically applicable doses, scopolamine exerts prominent CNS effects at low therapeutic doses. This difference may be explained by a better penetration of scopolamine through the blood brain barrier. Adverse effects associated with the use of scopolamine can, in the majority of cases, be attributed to an extension of its pharmacodynamic effects, and result from excessive anticholinergic activity (Renner et al., 2005; Nachum et al., 2006).

The commonest side effects reported for scopolamine therapy are sedation, dry mouth, blurry vision, central cholinergic syndrome, and confusion (Apfel et al., 2010). Scopolamine produces mydriasis and cycloplegia by paralyzing the sphincter muscle of the iris and the ciliary muscle of the lens. These effects on eye may last up to 7–12 days after a topical application of scopolamine. Although, systematically administered scopolamine has little effect on the intraocular pressure, patients with narrow-angle glaucoma may develop dangerous increases in the intraocular pressure following scopolamine (Renner et al., 2005). Additional side effects include reduction of gastric secretions and salivation, decreases in smooth muscle tone in the gastrointestinal tract, leading to a hypomotility, dryness of the nasopharynx, mouth, bronchi, and bronchioli. Following smooth muscle relaxation after scopolamine administration, the airway resistance in the respiratory tract is reduced. The drug exerts a similar relaxing effect on the smooth muscle tone of urethra and bladder (Renner et al., 2005).

## CLINICAL EFFICACY

Transdermal delivery system patches have proved to be highly effective in the management of motion sickness and PONV. This is also supported by experimental data and studies on human volunteers, showing a higher efficacy of TDS compared to placebo (Table 1; Graybiel, 1979; Graybiel et al., 1981, 1982; Dahl et al., 1984; Shojaku et al., 1993). Kotelko et al. (1989) studied patients undergoing elective cesarean delivery under epidural anesthesia with the addition of epidural morphine for postoperative analgesia. They reported a significant reduction in nausea, vomiting, and retching when comparing the TDS group vs. placebo during the study period of 2–10 h after surgery. Additionally, the TDS group required less antiemetic medication during the first 24 postoperative hours. The adverse effects experienced by the patients in both groups were dizziness (8%), blurred vision (4% in TDS group vs. 2% placebo group) and disorientation (1% TDS group vs. none in the placebo group). As it was shown, side effects were minimal in both groups (Kotelko et al., 1989). Harnett et al. (2007) carried out a similar study in women undergoing cesarean delivery under spinal anesthesia and induced opioid analgesia to compare the effects of TDS, placebo, and ondansetron on PONV. They administered antiemetic prophylaxis after clamping the umbilical cord instead of applying the TDS before surgery as Kotelko et al. (1989) reported. This relevant difference should be considered in order to avoid potential exposure of the fetus and consequent side effects. Their results showed that TDS was significantly superior to ondansetron or placebo as a PONV prophylactic therapy. The overall postoperative emesis rate was 59.3% in the placebo group and was reduced to 40% in the scopolamine group and 41.8% in the ondansetron group, respectively. The authors evaluated the effects of aforementioned drugs at different time periods postoperatively (0–2 h, 2–6 h, and 6–24 h), and it was concluded that the efficacy of scopolamine was higher compared to ondansetron or placebo during the postoperative 6–24 h interval. The side effects reported during the study did not lead to discontinuation of drug use in any patient. Dizziness varied significantly among the groups, but the relative incidence was not consistent across the time intervals. Dry mouth was somewhat more common in the scopolamine group in the 6–24 h interval (9% in placebo group, 4% in ondansetron group, 19% in scopolamine group). Similarly, blurry vision was more common in the scopolamine group than placebo at 6–24 h (6% vs. 0%). Lethargy occurred in < 10% of subjects during all time intervals, and its frequency did not differ among the groups. The differences in drug effects at different time periods may be related to the pharmacokinetic and pharmacodynamic properties of the drug (Harnett et al., 2007).

**Table 1:** Characteristics and overview of studies using transdermal scopolamine to prevent postoperative nausea and vomiting or post discharge nausea and vomiting.

Author	No. of patients	Population	Intervention	Conclusion
Kotelko et al. (1989)	203	Elective cesarean section	TDS vs. placebo prior anesthesia	TDS group had significantly less nausea, vomiting, and retching between 2 and 10 h after surgery. TDS group required less antiemetic medication.
Bailey et al. (1990)	138	Laparoscopy surgery under general anesthesia	TDS vs. placebo placed at least 8 h prior surgery	TDS group had less nausea, vomiting, and retching. Severe nausea and/or vomiting were present in 62% of the placebo group and only 37% on TDS group Additionally, patients in TDS group were also discharged from the hospital sooner
Stromberg et al. (1991)	201	General plastic or orthopedic surgery under general anesthesia	TDS or placebo placed at least 6 h prior surgery	Patients with TDS had less vomiting episodes than placebo group (21% TDS vs. 36 % placebo). Also, 35% of TDS group experienced nausea compared with 65% from placebo
Harris et al. (1991)	34	Gynecologic surgery with general anesthesia	TDS patch vs. placebo patch placed at PACU arrival	Patients with TDS had less severity of nausea between 2–24 h and less severity of vomiting during the first 2 h.
Reinhart et al. (1994)	39	Outpatient ear surgery	TDS vs. placebo placed 2 h prior surgery	TDS group had lower VAS nausea score and lower incidence of nausea (31 vs. 62%).
	60	Middle ear surgery	TDS vs. placebo patch	TDS group had lower incidence of nausea (10 vs. 27%) and vomiting (10 vs. 43%) than placebo group.
Honkavaara et al. (1995)	50	Outer ear surgery	TDS vs. placebo patch	TDS group had lower incidence of nausea (12 vs. 28%), retching (0 vs. 4%) and vomiting (16 vs. 48%,) than placebo group.
Honkavaara (1996)	56	Middle ear surgery	TDS vs. placebo patch	TDS group, 69% were free from emesis.
Tarkkila et al. (1995)	60	Arthroplasty surgery of lower extremity	Diazepam 5–15 mg PO vs. promethazine 10 mg PO vs. promethazine and TDS	60% of the patients with promethazine and TDS were totally free from PONV compared to those pre-medicated with diazepam (40%) or promethazine alone (30%). Promethazine together with TDS reduced significantly the incidence of nausea and vomiting
Jones et al. (2006)	56	Subjects with risk factors for PONV receiving general aesthesia	Ondansetron IV + TDS patch vs. Ondansetron IV + placebo patch	Scopolamine group had lower incidence of PONV, longer time to report nausea and longer time to report emesis
White et al. (2007)	150	Bariatric laparoscopic or plastic surgery	TDS + droperidol 1.25 mg IV (plastic surgery) vs. TDS + ondansetron 4 mg IV (laparoscopic surgery)	Premedication with TDS was effective as droperidol or ondansetron in preventing nausea and vomiting in the early and late period
Harnett et al. (2007)	240	Cesarean section under spinal anesthesia	TDS + Placebo IV or TDS placebo + ondansetron 2.4 mg IV or TDS placebo + placebo IV after clamping the umbilical cord	Overall emesis was 59.3% in the placebo group, 40% in the TDS group and 41.8% in the ondansetron group
Einarsson et al. (2008)	48	Gynecologic laparoscopic surgery	TDS vs. placebo patch placed prior night of surgery	Patients in the scopolamine group had significantly less incidence of nausea and vomiting during the first 24 h after surgery
Sah et al. (2009)	126	Outpatient plastic surgery	TDS + ondansentron 4 mg vs. Placebo patch + ondansentron 4 mg	Patients in the scopolamine group had significantly less postoperative nausea between 8 and 24 h after surgery
Gan et al. (2009)	618	Outpatient laparoscopic or breast augmentation surgery	TDS or placebo patch 2 h prior surgery + ondansetron 2–4 mg IV 2–5 min before induction	The combination of TDS plus ondansetron significantly reduced nausea and vomiting/retching compared with ondansetron alone 24 after surgery, but not at 48 h
Lee et al. (2010a)	120	Major orthopedic surgery under spinal anesthesia	Dexamethasone 8 mg IV or dexamethasone 8 mg IV + ramosetron 0.3 mg IV or dexamethasone 8 mg IV + TDS patch	The combination of TDS and dexamethasone group had lower rate of nausea compared to the other two groups (17.5 vs. 55.0 and 50.0%) and required less antiemetic (5 vs. 35.0 and 22.5%)
Lee et al. (2010b)	74	Uterine artery embolization	TDS or placebo patch 30–60 min before surgery + ondansetron 4 mg IV at the end of surgery	Lower level of nausea with those treated with scopolamine patch compared with placebo during the first 24 h after surgery, the difference was statistically significant
Green et al. (2012)	120	Elective surgical procedure with high PONV risk expected	TDS or placebo patch + aprepitant 40 mg PO at least 1 h prior surgery	No statistical significance between both groups

Einarsson et al. (2008) concluded that the TDS significantly reduces the incidence and severity of nausea and vomiting in the first 24 h after gynecologic laparoscopic surgery. They found a significantly higher rate of visual disturbances compared with the placebo group (45.8% vs. 8.3%). Symptoms of dry mouth were also slightly more common in the scopolamine group, but generally did not seem to be bothersome to study participants (87.5% vs. 79.2%; Einarsson et al., 2008). Jones et al. (2006) compared the efficacy of active TDS plus ondansetron with a placebo patch plus ondansetron in high-risk patients scheduled to receive a short duration general anesthesia for no longer than an hour. They found that patients receiving a combination of TDS and ondansetron reported fewer incidences (39%) of PONV compared to those who received ondansetron alone (75%). The most frequently reported side effect was headache, which has been attributed to ondansetron (Jones et al., 2006). White et al. (2007) compared a combination of TDS with droperidol vs. TDS with ondansetron in patient undergoing major laparoscopic surgeries, the study showed that both combinations were equally effective in preventing nausea and vomiting during the first 72 h of the post-operative period with a complete response of 41 and 51%, respectively (White et al., 2007).

Recently, a study by Sah et al. (2009) showed the efficacy of TDS plus ondansetron vs. placebo patch plus ondansetron on the incidence of PONV in 126 patients undergoing plastic surgery. A statistically significant reduction in postoperative nausea between 8 and 24 h in patients who received TDS was observed. During the first 4 h, no significant difference between the two groups was revealed, which may be attributed to ondansetron administration in both groups. The most common side effect was dry mouth in 70% of patients in the transdermal group compared with 63% in the placebo group. Visual disturbance was found in 15% of the TD group compared to 5% of patients in the placebo group. Sedation was common and notable in 40% of TD group vs. 33% of the placebo group, probably related to postoperative opioid use (Sah et al., 2009). Gan et al. (2009) confirmed the previous findings; they expanded the time of observed effectiveness of TDS with data collected from 0–48 h postoperatively and included the largest sample size to date. The study examined active TDS plus ondansetron compared with placebo patch plus ondansetron as a prophylactic treatment for PONV in 620 female patients undergoing outpatient laparoscopic or breast augmentation surgery. This study found a significant reduction in PONV incidence 24 h after surgery in the group receiving TDS plus ondansetron compared to the reference group that received placebo patch plus ondansetron. Despite the fact that TDS has a slow onset of action, this study showed that the clinical benefits are apparent when TDS is administered in combination with ondansetron 2 h before induction of anesthesia. In addition, this trial observed that the overall incidence of adverse events was less frequent in the group receiving TDS in combination with ondansetron compared with the group receiving ondansetron alone (36.7% vs. 49%; Gan et al., 2009). The combined use of TDS and dexamethasone by Lee et al. (2010a) showed that patients undergoing major orthopedic surgery using patient-controlled analgesia (PCA) was more effective in preventing PONV compared with dexamethasone alone or dexamethasone plus ramosetron (47.5% vs. 82.5% and 50.0%, respectively; Lee et al., 2010a). Limitations of this study include the lack of a control group without prophylaxis, the 24 h limits of patient evaluation after surgery, inability to evaluate the drug’s effects on the urinary function because of the presence of an indwelling urinary catheter (Lee et al., 2010a). Lee et al. (2010a) designed a similar randomized controlled trial that evaluated the efficacy of preventing nausea using TDS with ondansetron vs. ondansetron alone after uterine artery embolization (UAE). The overall incidence of nausea after UAE was low; there was a lower level of nausea in patients treated with TDS compared to the group that did not receive ondansetron during the first 24 h after embolization. Adverse events were more common with the TDS group, with two patients experiencing episodes of profound disorientation and 71% reporting substantial dry mouth. These results suggest that although the TDS provides moderate reduction of nausea, its use is associated with infrequent but notable episodes of patient disorientation.Therefore, the decision whether to use or not a TDS should be based on careful consideration of the potential benefits vs. the possibility of unwanted side effects for a given practice setting (Lee et al., 2010b). Green et al. (2012) compared aprepitant alone vs. aprepitant with scopolamine in patients undergoing elective surgical procedures and with two or more Apfel four-point risk factors. The study showed no difference in complete response (63% vs. 57%, *P* = 0.57) between both groups In addition, there was no difference between the numbers of patients who did not report any PONV those who used a rescue medication (Green et al., 2012). Clinical trials with TDS have consistently demonstrated its safety and efficacy as an antiemetic in many medical situations that frequently result in severe nausea and vomiting. In a meta-analysis study, Apfel et al. (2010) revealed a reduced risk of severe nausea and vomiting in patients receiving TDS compared to patients receiving placebo. TDS was shown to reduce the risk of postoperative nausea (relative risk, *RR* = 0.77; 95% CI, 0.61-0.98; *P* = 0.03) The most prevalent side effect registered at 24–48 h after surgery was the occurrence of visual disturbances (*RR* = 3.35; 95% CI, 1.78–6.32) (Apfel et al., 2010).

## SAFETY AND TOXICITY

Studies have consistently shown that the TDS is safe and well tolerated initially in patients treated for motion sickness. Later studies showed its efficacy for prophylaxis of PONV in patients undergoing various surgical procedures, including abdominal, and orthopedic. The most common side effects described throughout the different studies were dry mouth (~29%), dizziness (less than 8%), blurred vision (less than 4%), and disorientation (less than 1%; Package Insert, 2006). TDS is not recommended for administration in children and should be used with special caution in patients with pyloric obstruction, intestinal obstruction, impaired liver, and kidney function, obstructive urinary dysfunction, and glaucoma. The patch is contraindicated in patients hypersensitive to scopolamine and other belladonna alkaloids, as well as plaster allergies (Renner et al., 2005; Package Insert, 2006). In pregnant women, scopolamine should be administered only when potential benefits will overweight the risks to the fetus. Currently, there are not enough data to prove Scopolamine’s safety in pregnant or lactating mothers (Ayromlooi et al., 1980; Evens and Leopold, 1980; Briggs et al., 1994), although the drug is considered compatible with nursing and is not considered teratogenic. Scopolamine toxicity has been described in a newborn with symptoms such as tachycardia, fever, and lethargy (Renner et al., 2005).

## DISCUSSION

Despite the recognition of PONV as a contributing factor to postoperative morbidity, it still remains an actual medical problem necessitating a search for newer therapies. Current PONV risk stratification tools are helpful in providing a semi-quantitative risk assessment of PONV. Nevertheless, newer approaches and screening methods are to be developed to better reflect the patients’ and clinicians’ perception of PONV, provide a reliable means of high-risk patient selection, and reduce the overall impact of this complication on perioperative outcome. PONV pharmacotherapy and prophylaxis are based on monotherapy using a single antiemetic or combinations of several drugs. Multiple clinical trials have proven the safety and clinical efficacy of the TDS in treatment of PONV in various patient groups when used as a single drug or in combination with other drugs. TDS may be an effective method of PONV prophylaxis when applied within 2 h prior to surgery and anesthesia (Kotelko et al., 1989; Reinhart et al., 1994; Jones et al., 2006; White et al., 2007; Gan et al., 2009; Sah et al., 2009; Lee et al., 2010a, b; Green et al., 2012). This can be explained by the fast release and absorption of the loading dose in the inner layer of the patch.

The most recent consensus guidelines for the management of PONV address the issue of providing antiemetic therapy in those patients in whom prophylaxis has failed. They recommend that an antiemetic from a different pharmacologic class than the drug(s) administered for prophylaxis be given (Gan et al., 2014). They cite that the 5-HT3 antagonist class as being the only class that have been extensively studied for the treatment of existing PONV, and that this class should be administered prefererentially if not already given for prophylaxis. The guidelines also list alternative treatments to treat established PONV including dexamethasone, droperidol, or promethazine, and low dose propofol (Gan et al., 2014). If TDS is selected as a first-line agent for PONV prophylaxis medication, then this would leave more of the other drug classes available to treat established PONV if prophylaxis happens to fail. Due to the longer duration and pharmacokinetic profile, TDS may also be beneficial for prevention of PDNV, although further studies need to be performed to study this effect.

In conclusion, an effective management of PONV mandates understanding the pathophysiological mechanisms of PONV development and pharmacologic profiles of the applied medications, as well as their interaction with anesthetics, and other drugs used during the perioperative period. Effective reduction of the frequency and severity of PONV in various patient groups can be achieved with judicious use mono- or combination pharmacotherapy or non-pharmacological methods. Current scientific evidence indicates that TDS may be effectively used for prophylaxis and therapy of PONV.

## Conflict of Interest Statement

Sergio D. Bergese has a consulting agreement with Baxter Healthcare Corporation, active since 2010. TDS is licensed by Baxter Healthcare Corporation. Joseph V. Pergolizzi is a consult for Baxter Healthcare Corporation, Eisai Inc., Hospira and GlaxosmithKline.
